# Mitigating First-Cycle Capacity Losses in NMC811 via Lithicone Layers Grown by Molecular Layer Deposition

**DOI:** 10.1021/acsami.2c23158

**Published:** 2023-04-11

**Authors:** Konstantin Egorov, Wengao Zhao, Kristian Knemeyer, Alejandro Nico Filippin, Andrea Giraldo, Corsin Battaglia

**Affiliations:** † Empa, Swiss Federal Laboratories for Materials Science and Technology, 8600 Dübendorf, Switzerland; ‡ BASF Schweiz AG, 4005 Basel, Switzerland

**Keywords:** lithicone, alucone, CEI, lithium-ion battery, MLD, coating, NMC811

## Abstract

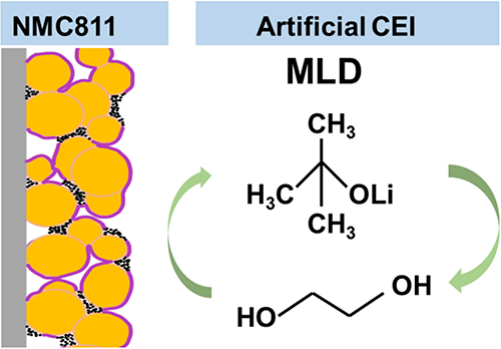

Nickel-rich LiNi_1–*x*–*y*_Mn_*x*_Co_*y*_O_2_ (NMC, 1 – *x* – *y* ≥ 0.8) is currently considered one of the most promising cathode materials for high-energy-density automotive lithium-ion batteries. Here, we show that capacity losses occurring in balanced NMC811||graphite cells can be mitigated by lithicone layers grown by molecular layer deposition directly onto porous NMC811 particle electrodes. Lithicone layers with a stoichiometry of LiOC_0.5_H_0.3_ as determined by elastic recoil detection analysis and a nominal thickness of 20 nm determined by ellipsometry on a flat reference substrate improve the overall NMC811||graphite cell capacity by ∼5% without negatively affecting the rate capability and long-term cycling stability.

## Introduction

Nobel laureate Wittingham and coauthors recently pointed out that the capacity and the energy density of lithium-ion batteries with LiNi_0.8_Mn_0.1_Co_0.1_O_2_ (NMC811) cathodes may be increased significantly by mitigating the so-called first-cycle capacity loss, which is neglected in many studies.^1^ The origin for this capacity loss is likely due to a combination of (1) loss of lithium due to irreversible reactions with the electrolyte, (2) loss of active cathode material due to irreversible structural changes, in particular on the surface of the cathode material, and (3) slow kinetics for lithium intercalation at high degrees of lithiation, i.e., close to the discharged state.^1,2^ In particular, with respect to (3), it was shown that a constant voltage step at the end of discharge can recover a significant fraction of the first-cycle capacity losses.

Here, we show that capacity losses associated with (1) and/or (2) can be mitigated by employing an artificial cathode electrolyte interphase (CEI) layer. We employ molecular layer deposition (MLD) to grow so-called lithicone layers^3^ directly onto porous NMC811 particle electrodes, thereby maintaining the electronic contact between NMC811 particles established by carbon black particles. MLD results in comparable conformality as atomic layer deposition (ALD), but typically achieves higher growth rates by replacing water as the oxidizing agent with ethylene glycol.^4,5^ Lithicone exhibits a moderate lithium-ion conductivity of 5 × 10^–8^ S/cm and low electronic conductivity and can be deposited at sufficiently low temperature to avoid thermal degradation of the polyvinylidene fluoride (PVDF) electrode binder. We demonstrate that lithicone is very efficient in reducing capacity losses during the first cycles resulting in an overall capacity increase of 5% during long-term cycling without negatively affecting the cell’s rate capability.

## Experimental Section

MLD layers were grown in an Arradiance GEMStar XT reactor equipped with an inductively coupled plasma source. The reactor is coupled to an argon-filled glovebox to allow transfer of samples without air exposure. Argon was used as a carrier gas for the MLD processes. MLD film growth was monitored *in situ* using a quartz crystal microbalance (QCM, Inficon) specifically designed for ALD/MLD. The backside of the quartz crystal is flushed with 40 sccm of argon to prevent unwanted film deposition at the backside of the crystal. The chamber is constantly flushed with 10 sccm of argon fed through precursor lines and 70 sccm of argon fed through the plasma source. Alucone layers were grown at 140 °C using trimethylaluminium (TMA, 98%, Strem) and ethylene glycol (EG, 99.8%, VWR Chemicals) as precursor. TMA and EG were kept at 40 and 65 °C, respectively, and the TMA and EG pulse times were set to 0.02 and 1.00 s, respectively. Lithicone layers were deposited at 135 °C with lithium *tert*-butoxide (LiOtBu, 98 + %, Strem) and EG as precursor. LiOtBu and EG were kept at 160 and 65 °C respectively, with pulse times of 0.02 and 1.00 s. The LiOtBu bottle was pressurized prior to each pulse to guarantee efficient vapor delivery to the reactor chamber as LiOtBu has a low vapor pressure.

Alucone and lithicone layers were characterized using ellipsometry (J.A. Woolam Co.) at an incidence angle of 60°. The Cauchy model was used to fit the data and extract layer thickness and the real part of the refractive index. Layer composition was determined using elastic recoil detection analysis (ERDA) using 13 MeV ^127^I ions and the Potku software^6^ to analyze ERDA spectra. ^6^Li and ^7^Li isotopes were treated separately because of the large relative difference in mass. Fourier-transform infrared spectroscopy (FTIR, Bruker Alpha) was performed in an argon-filled glovebox in attenuated total reflection (ATR) configuration.

Lithicone layer conformality was assessed using silicon wafer pieces with arrays of reactively etched holes, 2.5 μm width, and 157 μm depth, with a dead end (SmartMembranes). After lithicone layer growth, silicon wafer pieces were cut with a broad-argon-ion-beam miller (Hitachi IM4000 Plus) to image the lithicone layer on the walls of the holes by scanning electron microscopy (SEM, Hitachi S-4800).

Lithicone layers were also deposited on porous LiNi_0.8_Mn_0.1_Co_0.1_O_*x*_ (NMC811) particle electrodes with an areal capacity of 0.95 mAh/cm^2^, consisting of 90 wt % NMC811, 5 wt % polyvinylidene fluoride (PVDF) binder (Arkema HSV900), and 5 wt % carbon black Imerys Super C65 tape cast onto a 15 μm-thick aluminum foil (MTI) followed by calendaring at 1000 bar. The NMC811 particles were synthesized in-house and described in detail elsewhere.^7^ NMC811 electrodes with an areal capacity of 0.95 mAh/cm^2^ were punched into discs with 12 mm diameter and assembled against graphite electrodes (Customcells) with an areal capacity of 1.10 mAh/cm^2^ and a diameter of 15 mm in a CR2032-type coin cells (MTI) using a porous polymer separator (Celgard 2400) with a diameter of 16 mm and 35 mL of 1 M lithium hexafluorophosphate (LiPF_6_) in (3:7) ethylene carbonate (EC):ethyl methyl carbonate (EMC) + 2 wt % vinylene carbonate (VC) (Solvionic) as the electrolyte. After assembly, the cells were immediately polarized to a cell voltage of 1.5 V to prevent anodic dissolution of the copper current collector and then left to rest for 12 h for electrolyte soaking. Cells were then cycled using a multichannel potentiostat (Biologic BCS-805) with the cells kept at 25 °C in a climate chamber (Binder). Each cell was subjected to one formation cycle to 4.4 V at a rate of C/10 prior to any other cycling protocol. A discharge rate of 1C corresponds nominally to 0.95 mA/cm^2^. Cycling was performed within a voltage range of 2.7 and 4.4 V. C-rate capability at C/10, C/5, C/3, 1C, and 3C was assessed after the formation cycle without voltage hold at the upper cut-off voltage. Long-term cycling stability at C/3 was assessed after the formation cycle maintaining the cells in each cycle at the upper cut-off voltage until the current drops below C/20. Key performance parameters are given with error bars that were estimated as one standard deviation calculated over three cells for the coated electrodes and over eight cells for the pristine, i.e., non-coated, electrodes.

## Results and Discussion

**Figure 1:**
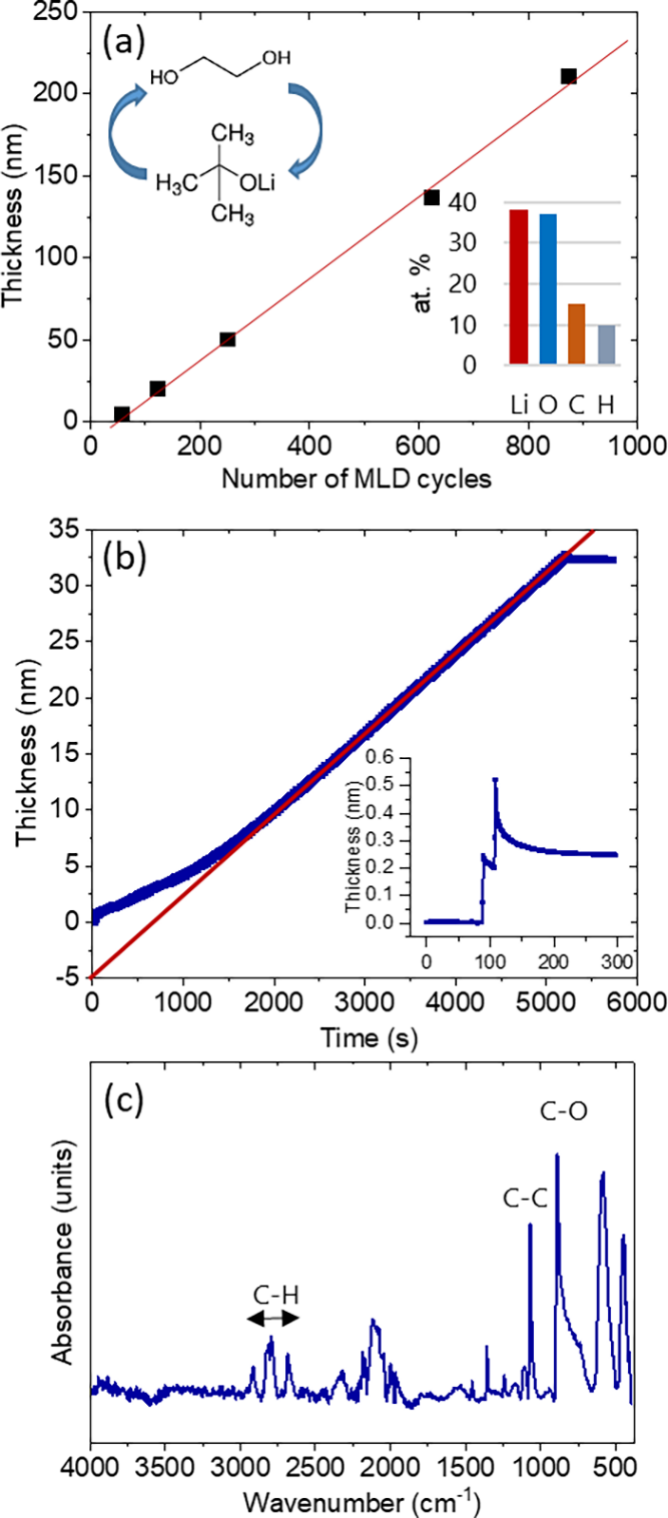
(a) Lithicone thickness *vs* the number of MLD cycles determined by ellipsometry. The inset shows the lithicone composition as determined by ERDA. (b) Lithicone thickness *vs* deposition time determined by QCM along with a fit to the data. The inset shows the QCM data recorded for a single lithicone deposition cycle. (c) ATR-FTIR spectrum of MLD lithicone layer grown on a silicon wafer.

Figure 1a shows the thickness of the lithicone layer as determined by ellipsometry on a flat silicon wafer as a function of the MLD cycle number. The lithicone layer thickness grows linearly with the number of MLD cycles after ∼50 “nucleation cycles” during which no lithicone layer growth is detected by ellipsometry. The growth rate extracted from the slope of the linear fit is about 2.5 Å/cycle, which is close to the previously reported value for lithicone layer growth at 135 °C.^3^ The refractive index determined by ellipsometry is 1.46. The lithicone layer composition as determined by elastic recoil detection analysis (ERDA) is given in the inset of Figure 1a, with the corresponding depth profiles provided in Figure S1. The lithicone layer consists of 38 at. % Li, 37 at. % O, 15 at. % C, and 10 at. % H resulting in a layer stoichiometry of LiOC_0.4_H_0.3_. The hydrogen-to-carbon ratio is lower than what would be expected from EG.

Lithicone layer growth as monitored by QCM is shown in Figure 1b. The QCM surface was precoated with a 10 nm-thick thermal ALD Al_2_O_3_ layer prior to lithicone deposition to start from a well-defined surface chemical state. Consistent with the ellipsometry data in Figure 1a, we also observe a linear mass growth with MLD cycles by QCM after a “nucleation period” of 1200 s with a lower deposition rate corresponding to 25 cycles. The inset of Figure 1b shows QCM data recorded during steady growth conditions for a single lithicone deposition cycle. The mass gain contributions from LiOtBu and EG are clearly resolved.

An ATR-FTIR spectrum measured on a 100 nm-thick lithicone layer grown on a silicon wafer piece is shown in Figure 1c. The strong peaks at 1066 and 1092 cm^–1^ are attributed to EG symmetric and antisymmetric C–O stretching vibrations that were also observed for alucone.^8^ The small peak at 1241 cm^–1^ is also associated with the C–O stretching vibration. The peaks at 1357 and 1453 cm^–1^ are attributed to CH_2_ twist, CH_2_ wag, CH_2_ scissor, and COH vibrations and were also reported for alucone.^8^ A strong set of peaks in the range of 2850–3000 cm^–1^ is attributed to C–H stretching vibrations.^8,9^ No peaks were found at >3000 cm^–1^ indicating typically an absence of O–H groups in the lithicone layer. The absence of those peaks was also reported for lithicone layers grown using lithium bis(trimethylsilyl)amide (LiHMDS) and EG.^9^ However, the authors of this study found that C–H peaks below 3000 cm^–1^ are dampened when the lithicone layer was exposed to CO_2_ during the deposition and thus came to the conclusion that the O–H peaks are superimposed with C–H peaks below 3000 cm^–1^.^9^

**Figure 2:**
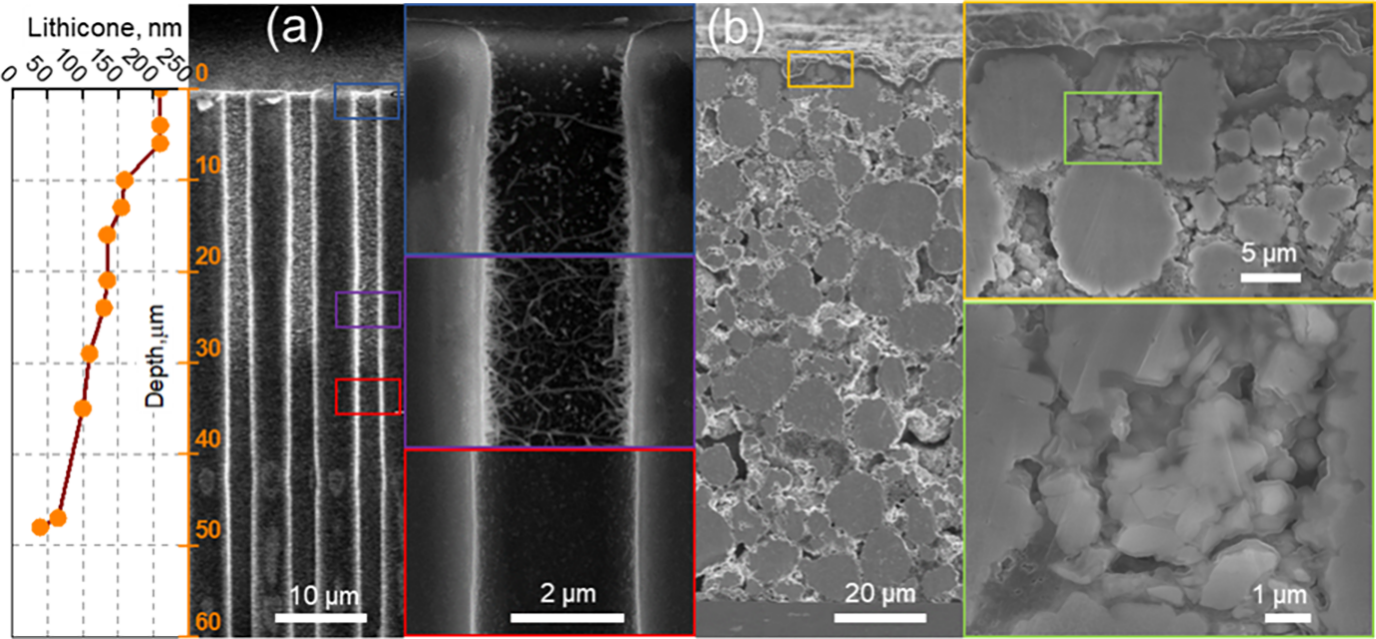
(a) SEM image of an ion-milled cross section across silicon wafer with etched holes coated with a nominally 200 nm-thick lithicone layer, lithicone layer thickness evolution with hole depth is shown to the left, (b) SEM image of ion-milled cross section across a 100 μm-thick NMC electrode coated with a nominally 200 nm thick lithicone layer, images with higher magnification are shown to the right.

Figure 2a shows a secondary-electron SEM image of an ion-milled cross section across the silicon wafer with the 2.5 μm-wide and 157 μm-deep etched holes into which a lithicone layer with a nominal thickness of 200 nm was deposited. The layer thickness at the top of the holes is about 210 nm and then monotonously decreases when going deeper into the holes. The minimal thickness that can be resolved is about 30–40 nm at a depth of 48 μm.

Figure 2b shows an analogous SEM image of a cross section across a 100 μm-thick porous NMC811 electrode with an areal capacity of 4.6 mAh/cm^2^ and high tortuosity onto which a 200 nm-thick lithicone layer was deposited. Although the NMC811 particles are not precoated, they are in contact with the PVDF binder and carbon black particles, as can be seen from the images in Figures S2 and S3, the former guaranteeing the mechanical integrity of the electrode and the latter establishing electronic contact between NMC811 particles. NMC811 particles near the top of the electrode are well coated with lithicone. However, it is difficult to distinguish the lithicone layer on the NMC811 particles from the lithicone layer that grows on the carbon black particles. As a result, lithicone forms a dense layer clogging small pores between agglomerated carbon black particles as shown in Figure S4. Clogging of these small pores between carbon black particles reduces the ability of MLD to cover the internal surfaces of the NMC electrode. Nevertheless, visible coverage is obtained to a depth on the order of 5–10 μm as can be seen from Figure 2b, corresponding approximately to the coating of the first NMC811 particle layer in the electrode. Coating of NMC811 particles in lower lying layers may occur, but is challenging to resolve in SEM cross-sectional images.

The electrochemical performance of lithicone-coated NMC811 electrodes with an areal capacity of 0.95 mAh/cm^2^ corresponding to an electrode thickness of about 15 μm was investigated in NM811||graphite coin cells. NMC811 electrodes with a nominally 10 and 20 nm-thick lithicone layer, as well as a pristine reference electrode without coating and a second reference electrode with a 10 nm-thick alucone layer were compared.

**Figure 3:**
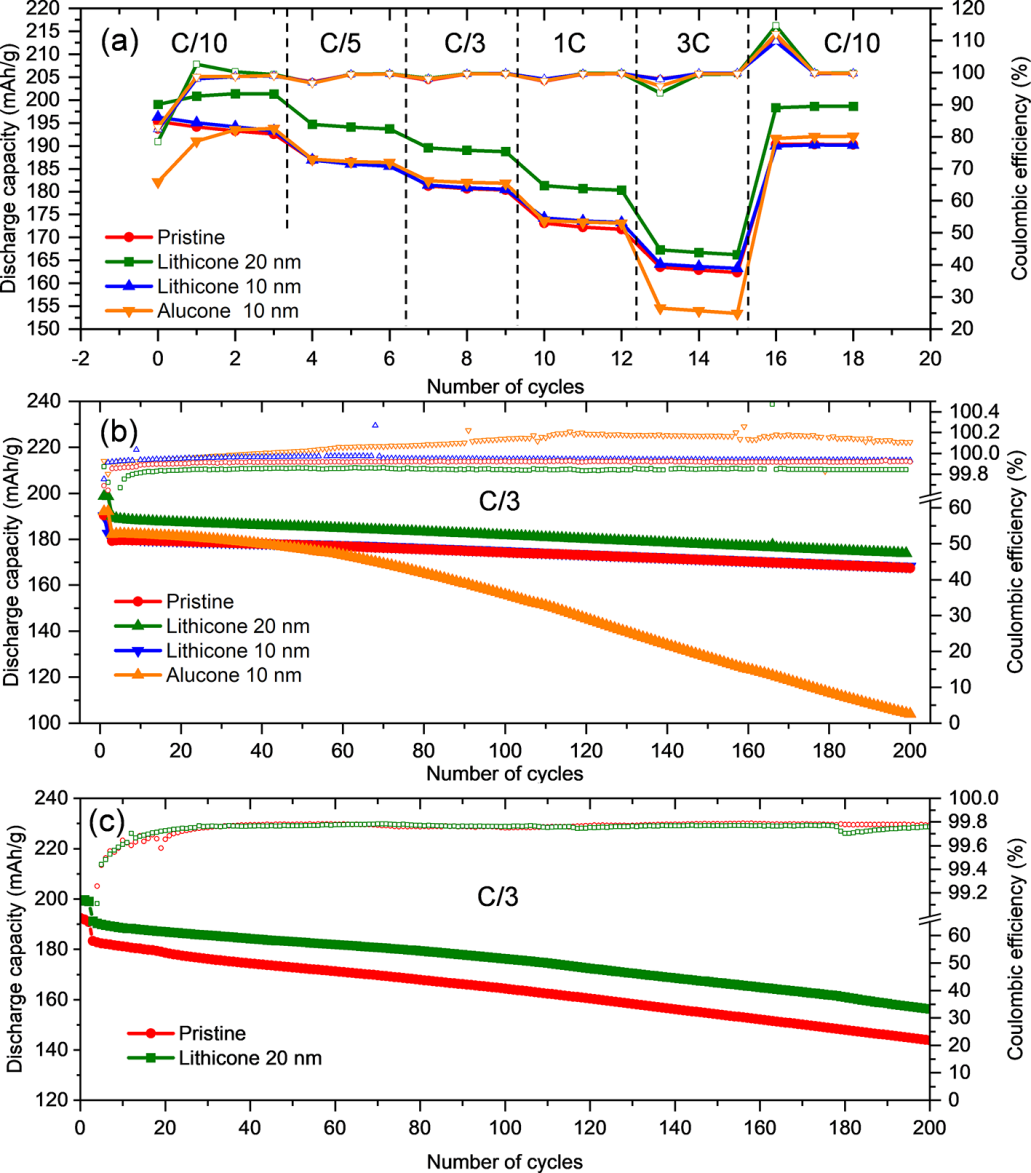
(a) Formation cycles and C-rate test of NM811||graphite coin cells. (b) Long-term cycling at C/3 at 25 °C. (c) Accelerated aging during long-term cycling at 40 °C.

Figure 3a shows the discharge capacity of the formation cycle at C/10 followed by discharge capacities obtained during the C-rate test consisting of 3 cycles at C/10, C/5, C/3, 1C, 3C, and C/10. The electrode with 20 nm lithicone shows the best initial discharge capacity of 199 mAh/g and maintains on average ∼5% higher capacity at all C rates, while the pristine electrode and the electrode with 10 nm lithicone reach only 194 mAh/g initial capacity (see also Table 1).

**Table 1:** Discharge Capacities and Coulombic Efficiency Extracted from Figure 3

	pristine	alucone 10 nm	lithicone 10 nm	lithicone 20 nm
first cycle capacity, mAh/g	194 ± 2	183 ± 4	195 ± 2	199 ± 2
first cycle Coulombic efficiency, %	81.7 ± 0.4	78.4 ± 0.5	82.2 ± 0.3	82.8 ± 0.3
capacity at C/10, mAh/g	191 ± 2	194 ± 2	192 ± 2	201 ± 2
capacity at 3C, mAh/g	161 ± 2	154 ± 3	161 ± 2	167 ± 2
capacity at 200th cycle, mAh/g	167 ± 2	110 ± 20	169 ± 2	174 ± 2

Although the electrode with 20 nm lithicone and the pristine electrode show approximately the same first-cycle charge capacity of 240 mAh/g (Figures S5 and S6), the electrode with 20 nm lithicone shows a significantly higher second-cycle charge capacity compared to the pristine electrode (Figure S5), indicating that lithicone reduces capacity losses during the first cycle by reducing the consumption of active lithium and electrolyte decomposition during CEI formation, especially during the first cycle.

The lowest initial discharge capacity is obtained with the electrode with 10 nm alucone reaching only 183 mAh/g. However, already during the third cycle, the capacity recovers and is comparable to the capacity obtained for the pristine electrode. We speculate that this phenomenon may be related to alucone lithiation improving alucone wettability by the electrolyte (see discussion below).

During long-term cycling shown in Figure 3b, the electrode with 20 nm lithicone maintains ∼5% higher capacity compared to the pristine electrode and the electrode with 10 nm lithicone with relatively high capacity retention >90% after 200 cycles. In contrast, the electrode with 10 nm alucone undergoes rapid capacity fading with a capacity retention of only 55% after 200 cycles. The Coulombic efficiency of this cell is >100%, indicating parasitic reactions, which lead to a rapid capacity fading.

In conclusion, the 20 nm lithicone layer results in a higher capacity during the formation cycles. The cell then maintains this additional capacity during the rate test and long-term cycling compared to the cell with the pristine electrode. We attribute the similar cycling performance of the cell with the pristine electrode and the cell with the electrode with 10 nm lithicone to the delayed nucleation of lithicone on NMC811 compared to the reference silicon wafer piece onto which lithicone was co-deposited to assess layer thickness. Thus, the lithicone layers on NMC811 may be thinner than on the silicon reference wafer pieces.

Accelerated aging was performed at 40 °C and is shown in Figure 3c. While both cells exhibit more pronounced capacity fading, the cell with 20 nm lithicone exhibits again a higher capacity during the first cycle and maintains this higher capacity compared to the cell with the uncoated electrode.

**Figure 4:**
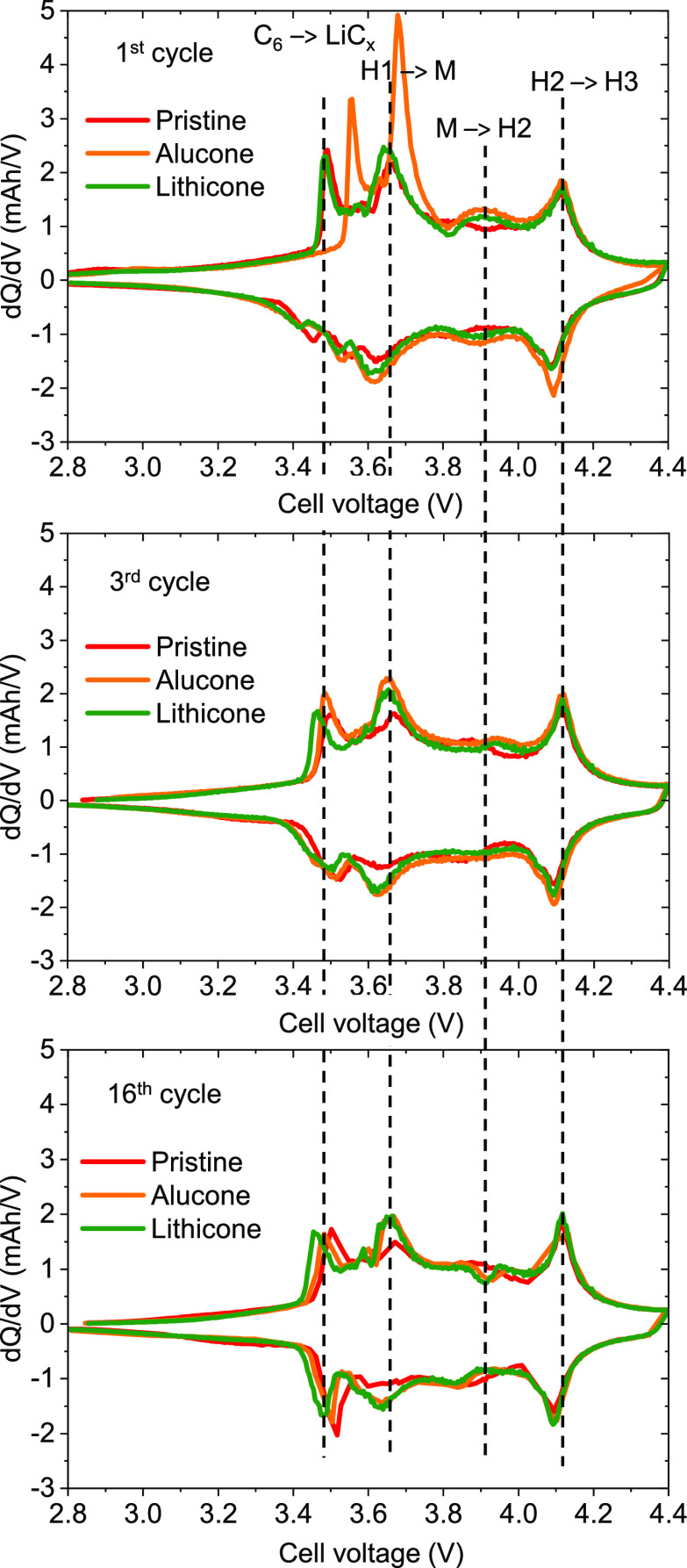
dQ/dV *vs* cell voltage during the (a) 1st cycle, (b) 3rd cycle, (c) and 16th cycle at C/10 for pristine NMC811 and lithicone- and alucone-coated NMC811.

Figure 4a shows the dQ/dV plots of the 1st, 3rd, and 16th cycles of the cells cycled at C/10 with the pristine, 20 nm lithicone-coated, and 10 nm alucone-coated electrodes. The peak at 3.5 V is ascribed to lithium intercalation into graphite.^10^ Peaks at higher cell voltage are attributed to the hexagonal to monoclinic (H1 to M) phase transition of NMC811 at 3.7 V,^10^ the monoclinic to hexagonal (M to H2) phase transition at 3.9 V, and the hexagonal to hexagonal (H2 to H3) phase transition at 4.1 V.^10^

Inspection of Figure 4 reveals that the two first peaks of the cell with the alucone layer occur at a 70 mV higher cell voltage during the first charging step compared to the two peaks of the cell with the pristine and the lithicone layer, indicating an additional overpotential associated with the not-yet lithiated alucone layer and significant charge transfer (see also voltage vs capacity curves in Figure S6). Interestingly, already during the first discharge, this shift is no longer observed, indicating lithiation (and possibly partial decomposition) of the alucone layer, resulting in a significant reduction of the overpotential.

For the pristine NMC811 electrode, the peak associated with lithium intercalation into graphite shifts to slightly higher cell voltages with an increasing cycle number. We attribute this peak shift to an additional overpotential caused by solid electrolyte interphase (SEI) formation on the graphite and/or cathode electrolyte interphase (CEI) formation on the NMC811 electrode. Interestingly, the opposite trend is observed for the cell with the lithicone-coated NMC811 electrode, indicating that lithicone acts as a benign artificial CEI.

The peaks associated with NMC811 phase transitions become smaller and tend to shift toward higher cell voltages with increasing cycle numbers for all cells, which is typical for NMC particle degradation^7,11,12^ (Figure S7). As pointed out before, the positive effect of lithicone can be ascribed to the higher Coulombic efficiency achieved during the first few cycles, which results in overall ∼5% higher capacity in successive cycles but does not significantly affect the aging behavior during long-term cycling.

## Conclusions

In this study, the impact of lithicone and alucone layers grown by molecular layer deposition on porous NMC811 particles electrodes was studied and assessed in NMC811||graphite coin cells. Cells with lithicone-coated NMC811 electrodes show higher initial capacity than cells with pristine NMC811 electrodes and maintain roughly 5% higher capacity during rate tests and long-term cycling, while relative capacity fading is comparable to the cells with pristine electrodes. In contrast, cells with alucone-coated NMC811 electrodes show lower initial capacity and a much more pronounced capacity fading. Our study demonstrates that lithicone is a promising material to mitigate capacity losses during formation cycling.

## Supporting Information Available

ERDA depth profile of 200 nm-thick lithicone films deposited on a silicon wafer; uncoated NMC811 cathode with an areal capacity of 4.6 mAh/cm^2^; close-up view of NMC811 particles surrounded by PVDF binder and carbon black particles; examples of open small porosity in between agglomerated carbon black particles and structure of lithicone layer near the electrode surface; charge and discharge capacities at C/10 *vs* cycle number for the cell with the pristine electrode and for the cell with the 20 nm lithicone electrode; cell voltage *vs* capacity during the formation cycle at C/10 for the cell with the pristine electrode, with the electrode with 20 nm lithicone, and with the electrode with 10 nm alucone; dQ/dV *vs* cell voltage at different charge/discharge cycles for pristine, alucone, and lithicone /coated cathodes; and Nyquist plots of the impedance spectra for the 20 nm lithicone and pristine electrodes (PDF)
