# Supplementary material for: Mitigating First-Cycle Capacity Losses in NMC811 via Lithicone Layers Grown by Molecular Layer Deposition
Source: ACS Appl Mater Interfaces. 2023 Apr 11;15(16):20075–80. doi: 10.1021/acsami.2c23158 (PMC10141247; doi:10.1021/acsami.2c23158)
Supplement: Supplementary file 1 — am2c23158_si_001.pdf [file am2c23158_si_001.pdf]

Supplementary Information

Mitigating first-cycle capacity losses in NMC811 via lithicone layers grown by molecular layer deposition

Konstantin Egorov[1], Wengao Zhao[1], Kristian Knemeyer[2], Alejandro Nico Filippin[2], Andrea Giraldo[2], Corsin Battaglia[1]

[1]Empa, Swiss Federal Laboratories for Materials Science and Technology, 8600 Dübendorf, Switzerland,

[2]BASF Schweiz AG, 4005 Basel, Switzerland

E-mail: konstantin.egorov@empa.ch

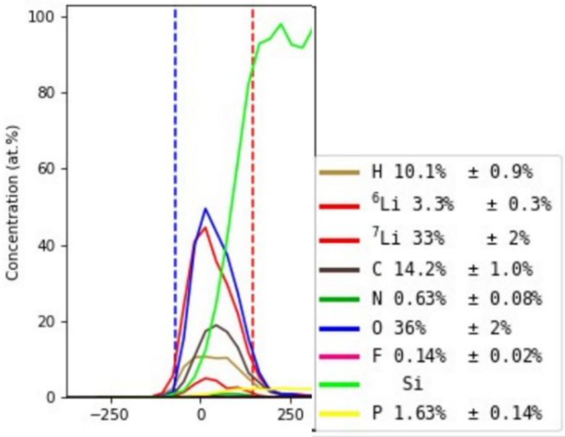

Figure S1: ERDA depth profile of a 200 nm thick lithicone films deposited on a silicon wafer. The carbon and hydrogen profiles are well distributed over the layer. No atmosphere contamination peak is observed.

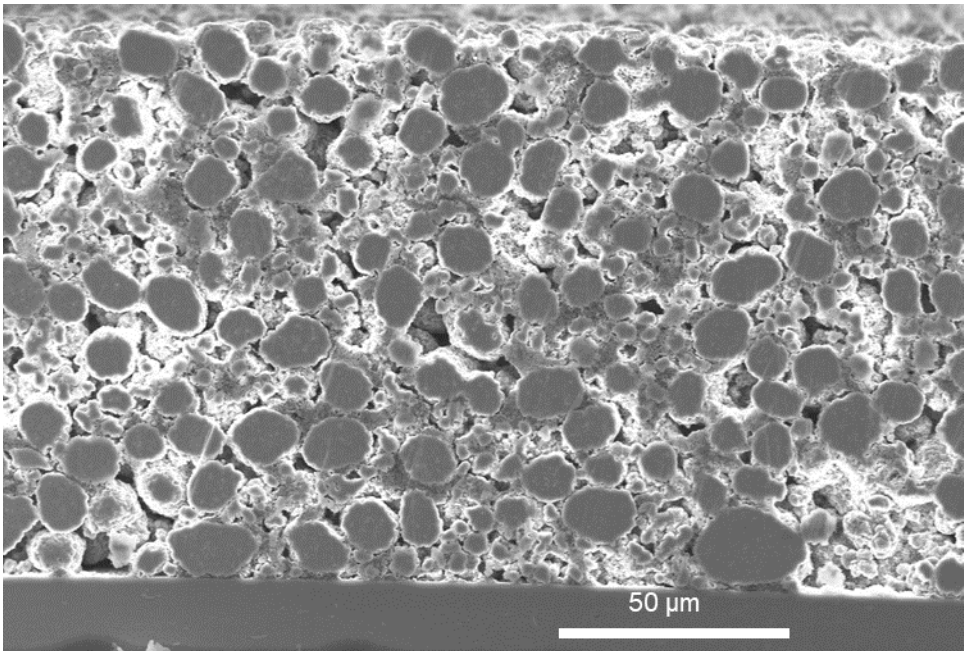

Figure S2: Uncoated NMC811 cathode with an areal capacity of 4.6 mAh/cm^2.

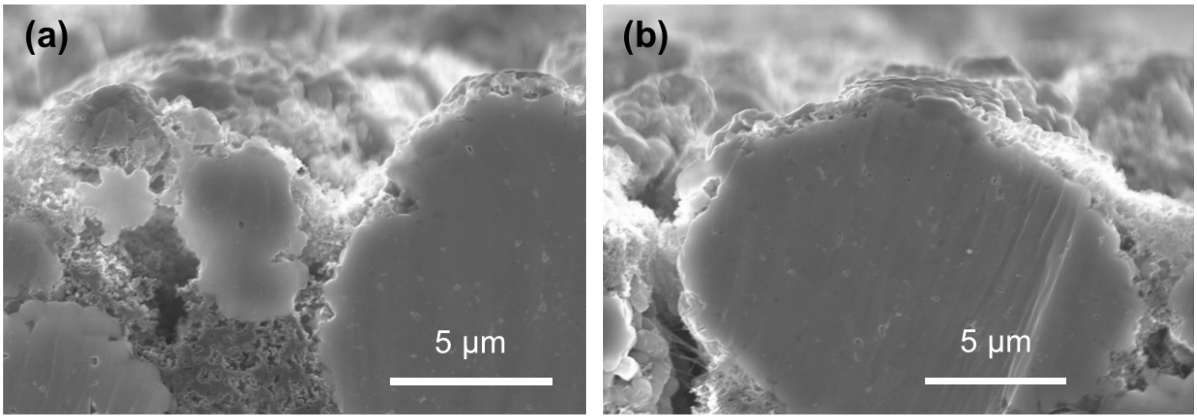

Figure S3: Close-up view of NMC811 particles surrounded by PVDF binder and carbon black particles (a) and (b).

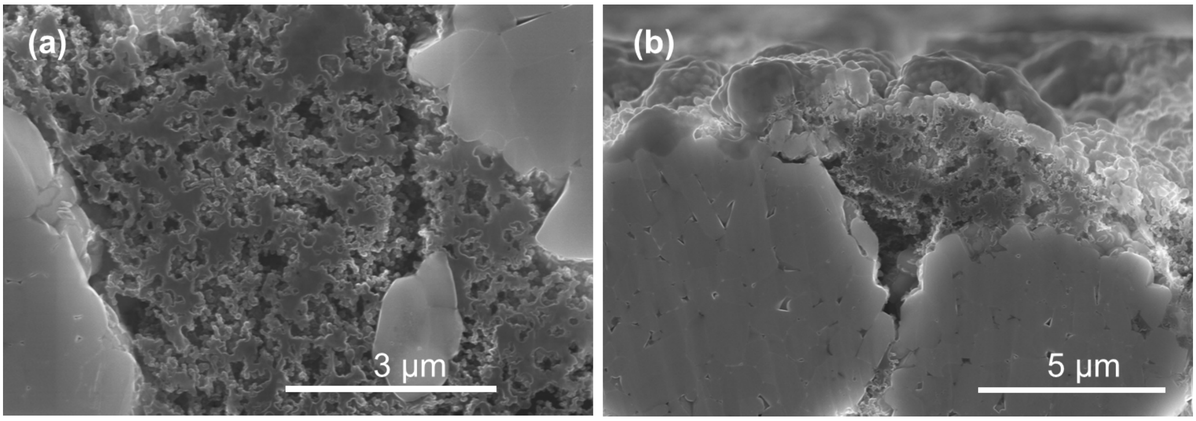

Figure S4: Examples of open small porosity in between agglomerated carbon black particles and structure of lithicone layer near the electrode surface (a) and (b).

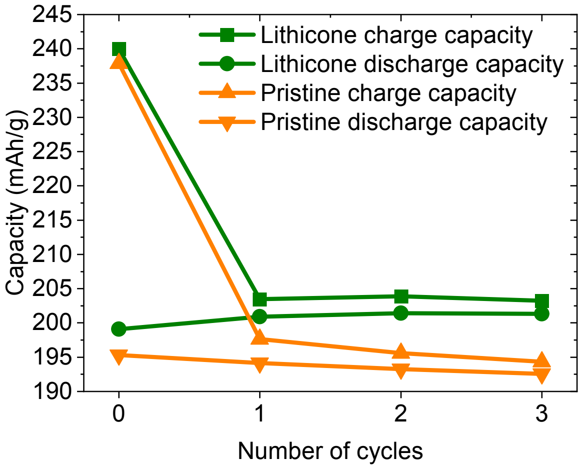

Figure S5: Charge and discharge capacities at C/10 *vs* cycle number for the cell with the pristine electrode and for the cell with the 20 nm lithicone electrode.

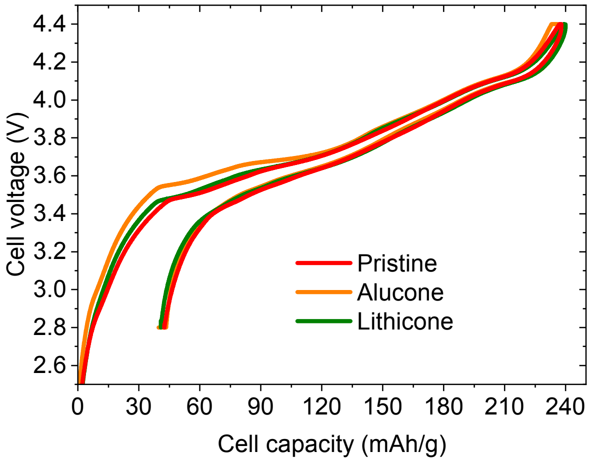

Figure S6 : Cell voltage *vs* capacity during the formation cycle at C/10 for the cell with the pristine electrode, with the electrode with 20 nm lithicone, and with the electrode with 10 nm alucone.

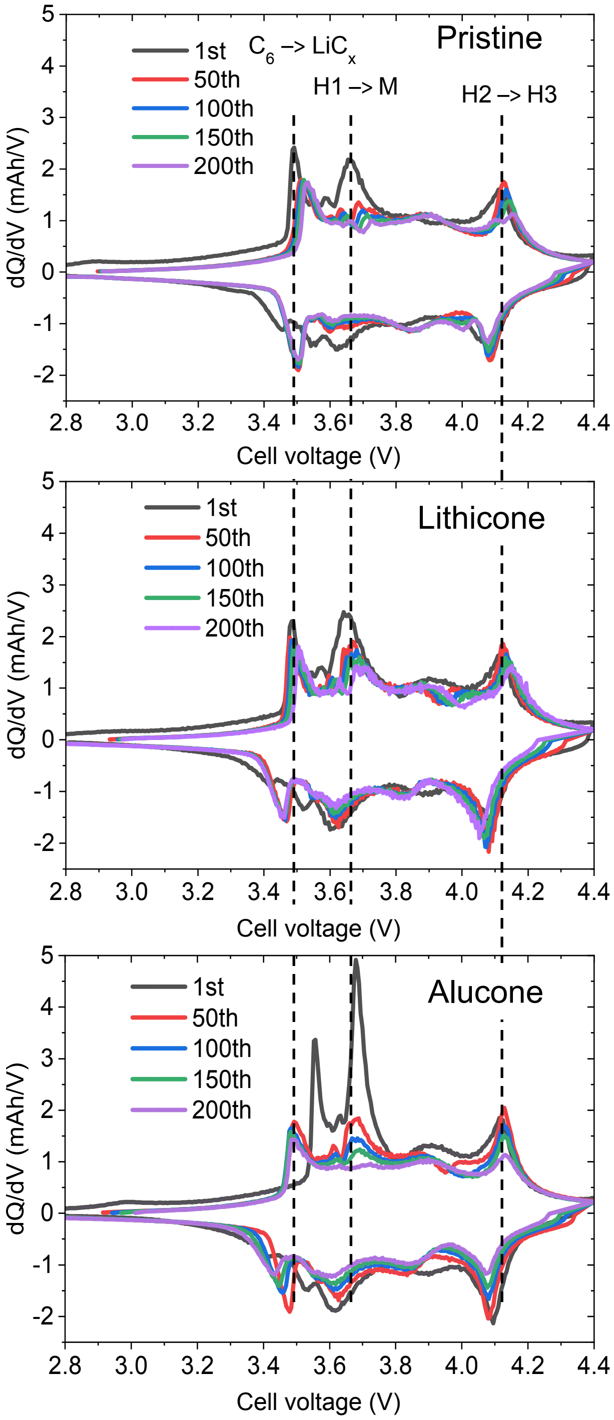

Figure S7: dQ/dV *vs* cell voltage at different charge/discharge cycles for pristine, alucone and lithicone /coated cathodes.

The EIS analysis was performed on CR2032-type coin cells with a multichannel potentiostat Biologic BCS-805. The cell was charged up to 4.3V and relaxed for 1 hour followed the 10 mV perturbation signal in the frequency range of 10^5–1 Hz was applied.

The initial EIS curves were collected for lithicone-coated and pristine cells. The significant differences were observed only at the mid-frequency (<20 Hz) region which is in full cells attributed to charge transfer resistance between electrode and electrolyte. Higher charge transfer resistance for lithicone-coated cell is attributed to the electronically isolative properties of lithicone. However, after the electrochemical formation cycles the resistances decrease significantly for both cells.

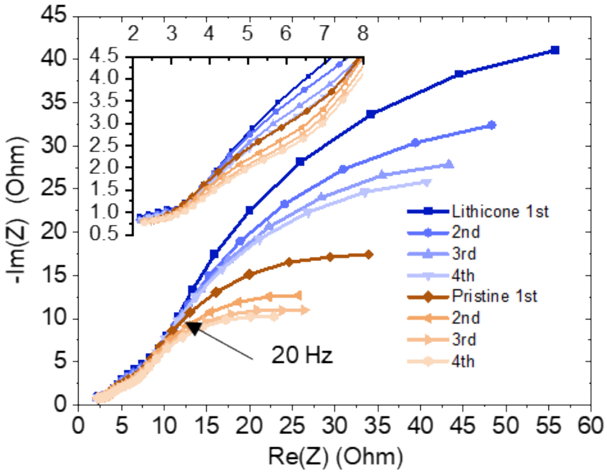

Figure S8 Nyquist plots of the impedance spectra for the 20 nm lithicone and pristine electrodes.
